# The correlation between the pathological characteristics of pulmonary invasive mucinous adenocarcinoma and radiomic features and abnormal expression of the FoxM1 and Sox9 genes

**DOI:** 10.1371/journal.pone.0345780

**Published:** 2026-04-09

**Authors:** Aizhu Sheng, Cheng Yan, Yizhai Ye, Zhenhua Yang, Bihua Wang

**Affiliations:** 1 Department of Radiology, Ningbo No.2 Hospital, Wenzhou Medical University, Ningbo, China; 2 Department of Radiology, Zhongshan Hospital, Fudan University, Shanghai, China; 3 Department of Radiology, Ninghai First Hospital, Ningbo, China; 4 Department of Thoracic Surgery, Ningbo No.2 Hospital, Wenzhou Medical University, Ningbo, China; 5 Department of Radiology, Tiantai Hospital of Traditional Chinese Medicine, Taizhou, China; Hokkaido University: Hokkaido Daigaku, JAPAN

## Abstract

**Objective:**

We aimed to analyze the correlation between radiomic features of pulmonary invasive mucinous adenocarcinoma (PIMA), abnormal expression of the FoxM1 and Sox9 genes, and pathological characteristics of the tumor.

**Methods:**

From June 2021 to October 2024, we selected 150 patients with PIMA and 150 patients without PIMA from Ningbo No.2 Hospital. CT radiomic parameters and the serum mRNA levels of FoxM1 and Sox9 were compared between the groups and correlated with pathological features.

**Results:**

Patients with PIMA exhibited higher Kurtosis and Entropy in CT radiomic features and increased mRNA levels of FoxM1 and Sox9 compared to patients without PIMA (*P* < 0.05). These parameters were negatively correlated with differentiation degree and positively correlated with TNM staging (*P* < 0.05). Kurtosis, Entropy, plasma FoxM1 mRNA, and Sox9 mRNA are all influencing factors of PIMA (*P* < 0.05). The combined AUC for differential diagnosis of PIMA was the highest for Kurtosis, Entropy, plasma FoxM1, and Sox9 mRNA levels.

**Conclusion:**

CT radiomic parameters and the plasma mRNA levels of FoxM1 and Sox9 among patients with PIMA were closely associated with tumor differentiation and TNM staging, offering invaluable references for the differential diagnosis of PIMA.

## 1. Introduction

Pulmonary invasive mucinous adenocarcinoma (PIMA) is a specific subtype of lung adenocarcinoma, which was first recognized by the 2011 International Multidisciplinary Classification of Lung adenocarcinoma. Although the incidence of PIMA is relatively low, accounting for 2%−10% of lung adenocarcinomas, the risk of malignancy is higher with PIMA than with non-PIMA [1]. There are significant differences between PIMA and non-PIMA in terms of cell types, cytological characteristics, metastasis modes, growth patterns, and treatment strategies. However, PIMA has no specific clinical presentation and laboratory examination, which makes its diagnosis and differentiation challenging. Although previous studies have shown that PIMA and non-PIMA have certain differences in CT image features, these features also lack specificity and suffer from high false positive rates or false negative rates [2]. Currently, puncture biopsy serves as the primary method for pathological diagnosis. Although it has high accuracy, this procedure relies on invasive operations guided by CT, ultrasound, and bronchoscopy, making it unacceptable for some patients [3]. Therefore, it is of great significance to find a non-invasive and efficient diagnostic technique for the differential diagnosis of PIMA.

Significant progress has been made in the differentiation, staging, differentiation assessment, and prognosis prediction of benign and malignant tumors by extracting massive texture parameters from traditional medical images and combining them with automated algorithms, transforming them into quantitative information that can be analyzed [4]. Recently, serological studies have shown that some specific factors, such as forkhead transcription factor M1 (FoxM1) and sex-determining region Y-frame protein 9 (Sox9), are highly expressed in patients with lung adenocarcinoma, which is closely associated with pathological characteristics, such as the degree of differentiation and the clinical stage of tumors [5–6]. However, there is currently a lack of studies specifically investigating the relationship between CT radiomic features, plasma FoxM1 and Sox9 levels, and the pathological characteristics of PIMA. The potential application value of these factors in the non-invasive differential diagnosis of PIMA remains to be further explored.

Therefore, this study aimed to systematically explore the correlation between the imaging characteristics of PIMA, abnormal expression of FoxM1 and Sox9, and the pathological features of tumors by combining imaging and serological indexes. This study provides a new idea for the non-invasive diagnosis of PIMA and offers a scientific basis to formulate individualized treatment strategies.

## 2. Materials and methods

### 2.1. General information

150 patients with PIMA who were admitted to Ningbo No.2 Hospital from June 2021 to October 2024 were enrolled in the PIMA group, and 150 patients with invasive non-mucinous during the same period were enrolled in the non-PIMA group. The inclusion criteria were as follows: 1) lung adenocarcinoma meeting the diagnostic criteria of Chinese Guidelines for the Diagnosis and Treatment of Primary Lung Cancer (2015 Edition) [7]; 2) PIMA and invasive non-mucinous adenocarcinoma were confirmed by clinical and pathological assessment; and 3) the image quality was good and did not affect the interpretation of the results. The tumor diameter in the PIMA group was > 3 cm, while no strict tumor size restriction was applied in the non-PIMA group.The exclusion criteria were as follows: 1) coexistence of other malignant tumors, autoimmune diseases, congenital lung diseases, or anatomic abnormalities of the respiratory tract; 2) before enrollment, the patients underwent surgery, chemoradiotherapy, or other treatments. 3) psychiatric or therapeutic abnormalities; 4) hematologic diseases; 5) pregnant or lactating women. General data were comparable between the two groups (*P* > 0.05) (Table 1).

**Table 1:** Comparison of general information between the two groups n(%)/($\overset{―}{x}$±s).

Group	n	male/female	age(year)	Smoking	TNM stage		Tumor Diameter (cm)
					Ⅰ ~ Ⅱ	Ⅲ	
PIMA	150	85/65	65.49 ± 8.74	87(58.00)	82(54.67)	68(45.33)	4.15 ± 0.80
non-PIMA	150	92/58	63.86 ± 7.91	72(48.00)	76(50.67)	74(49.33)	3.77 ± 1.05
*t*/*χ*^*2*^		0.675	1.694	3.011	0.481		1.361
*P*		0.411	0.091	0.083	0.488		0.175

All procedures involving human participants in this study were conducted in accordance with the ethical standards of the National Research Committee and the 1964 Helsinki Declaration, along with its later amendments or comparable ethical standards. The study was approved by the Ethics Committee of Ningbo No.2 Hospital (Approval No. YJ-KYSB-NBEY-2020-191-01).Given the retrospective nature of the study and the use of anonymized data, the requirement for informed consent was waived by the Ethics Committee.

### 2.2. Methods

#### 2.2.1. CT examination method.

All enrolled patients underwent contrast-enhanced chest computed tomography (CT) scans using a Siemens Somatom Sensation 64-slice spiral CT scanner (Siemens Healthineers, Germany). The scanning range extended from the lung apex to the upper pole of both kidneys. The scanning parameters were set as follows: tube voltage 120 kV, tube current 90–60 mA, slice thickness 5 mm,pitch 1.0, and matrix 512 × 512. The non-ionic contrast agent iohexol was administered via the antecubital vein at an injection rate of 2.5–3.0 mL/s. Arterial and venous phase scans were performed at 25–30 seconds and 55–60 seconds after injection, respectively. The images were reconstructed with a slice thickness and interval of 1.0 mm and uploaded to a workstation.

CT images were manually segmented using image processing software to delineate regions of interest (ROIs) for subsequent texture analysis. All CT images were resampled to a uniform voxel size (1.0 mm × 1.0 mm × 1.0 mm), and grayscale normalization was performed using Z-score standardization to minimize the influence of variations in scanning protocols on feature stability.The original data was imported into the Omni-Kinetics software in DICOM format. Two senior clinical radiologists (with ≥8 years of experience) independently reviewed the images in a blinded manner, manually segmenting the lesions and delineating the regions of interest. The largest cross-section of the tumor was selected, avoiding areas of hemorrhage, necrosis, and cystic changes. The CT images were processed using linear interpolation and resampling (with isotropic voxel size of 1.0 × 1.0 × 1.0 mm³), followed by Z-score normalization to reduce feature dimensionality differences. After normalization, the fixed bin width method was used for discretization, with the bin width set to 25 HU to primarily calculate texture features. To capture multi-scale texture features, Laplacian of Gaussian (LoG) filtering was applied (with σ values set to 1.0 mm, 2.0 mm, and 3.0 mm), and the PyRadiomics software package was used to process the original images and the filtered images for texture analysis extraction.

Laws texture features were applied to characterize the texture patterns within the delineated ROIs. The following texture parameters were automatically extracted: Mean Value, Standard Deviation (std. Deviation), 10th Quantile (Quantile10), 25th Quantile (Quantile25), 50th Quantile (Quantile50), 75th Quantile (Quantile75), 90th Quantile (Quantile90), Skewness, Kurtosis, Entropy, Energy, Variance, Surface Area, Flatness, Elongation, Minor Axis, Major Axis, and High Gray Level Emphasis.

#### 2.2.2. Detection of the plasma mRNA levels of FoxM1 and Sox9.

On admission, 5 ml of venous blood was collected from patients in the two groups, and the supernatant was collected after centrifugation. Real-time fluorescence quantitative PCR was employed to determine the mRNA levels of FoxM1 and Sox9, and plasma total RNA was extracted following the operation procedure of the Trizol kit (Thermo Fisher Scientific, USA), and its concentration and purity were determined. cDNA template was synthesized using the reverse transcription kit (Thermo Fisher Scientific, USA). The reaction system was configured following the operation of the SYBR Green kit (Thermo Fisher Scientific, USA), and then an amplification reaction was conducted. The expression levels of FoxM1 mRNA and Sox9 mRNA were calculated using the 2-^ΔΔCt^, and GAPDH was employed as the internal reference. The forward primer for FoxM1 was 5’-GCAGCAGAAACGACCGAATC-3’, and the reverse primer was 5’-GGTCTTGGGGTGGGAGATTG-3’.

The primers were designed with Primer–BLAST, based on the human FoxM1 mRNA reference sequence (NCBI Accession No. XM_047428547.1).The forward primer for Sox9 mRNA was 5’-ATGAATCTCCTGGACCCCTTCA-3’, and its reverse primer was 5’-AGGTCGAGTGAGCTGTGTGTAGAC-3’. As for GAPDH, the forward primer was 5’-CATGTTCGTcATGGGTGTG-3’, and the reverse primer was 5’-GGTGCTAAGCAGTTGGTGGTG-3’.

#### 2.2.3. Pathological features.

The clinicopathological features of the PIMA group were analyzed, including gender, tumor diameter, age, TNM stage, differentiation degree, and lymph node metastasis.

### 2.3. Observation indicators

(1) Comparison of CT radiomics parameters and plasma FoxM1 and Sox9 mRNA levels between the two groups. (2) Comparison of CT radiomics parameters, plasma FoxM1 mRNA levels, and Sox9 mRNA levels among PIMA patients with different pathological features. (3) Correlation between CT radiomics parameters, plasma FoxM1 mRNA levels, Sox9 mRNA levels, and pathological features. (4) Diagnostic value of CT radiomics parameters, plasma FoxM1 mRNA levels, and Sox9 mRNA levels in the differential diagnosis of PIMA.

### 2.4. Follow-up

All enrolled patients were followed up to record postoperative recurrence and distant metastasis. Recurrence was defined as the emergence of new lesions at the primary site or regional lymph nodes detected via imaging examinations. Metastasis was defined as the appearance of new lesions in distant organs. The follow-up period ended in June 2025.

### 2.5. Statistical analysis

Data were analyzed using SPSS 25.0 software. Measurement data that passed the Shapiro–Wilk normality test and Levene’s test for homogeneity of variances were expressed as mean ± standard deviation ($\overset{―}{x}$ ± s). Comparisons between two groups were performed using the independent t-test, while comparisons among multiple groups were conducted using one-way analysis of variance (ANOVA), with within-group comparisons made using the LSD-t test. Enumeration data were expressed as n (%) and analyzed using the χ² test. Spearman correlation analysis was employed to examine the correlations between various indicators and pathological features. Multivariate logistic regression analysis (backward LR method) was used to identify independent diagnostic factors for PIMA. The receiver operating characteristic (ROC) curve and the area under the curve (AUC) were utilized to evaluate the diagnostic performance of individual indicators and combined diagnostic models. Differences in AUC values were compared using DeLong’s test. To assess the diagnostic efficacy of each indicator for PIMA, ROC curve analysis was performed with the PIMA group as positive samples and the non-PIMA group as negative samples. The AUC values for Kurtosis, Entropy, plasma FoxM1 mRNA, and Sox9 mRNA were calculated separately. A multivariate prediction model was further constructed using logistic regression, and a combined ROC curve was plotted. DeLong’s test was applied to compare the AUC values between individual and combined diagnostic methods. A *P*-value < 0.05 was considered statistically significant.

## 3. Results

### 3.1. CT image omics parameters of the two groups

**Fig 1:**
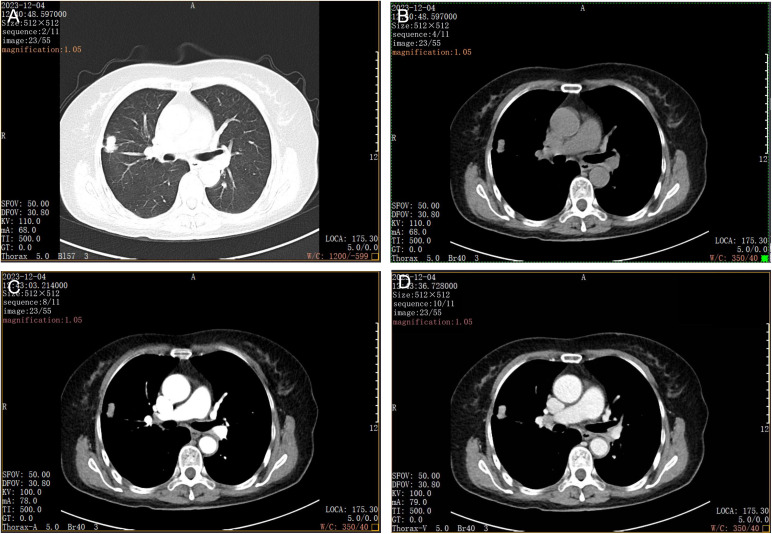
Typical imaging features of invasive mucinous adenocarcinoma of the lung. **A.** Scan lung, **B.**Scan mediastinum, **C.** Enhanced arterial phase, **D.** Enhanced arterial phase.

Initially, 18 radiomic features were extracted. After evaluation by intraclass correlation coefficient (ICC), 15 features with ICC > 0.75 were retained, including Mean Value, Standard Deviation, Quantile 10, Quantile 25, Quantile 50, Quantile 75, Quantile 90, Skewness, Kurtosis, Entropy, Variance, Surface Area, Flatness, Elongation, and High Gray Level Emphasis. Subsequently, least absolute shrinkage and selection operator (LASSO) regression (with λ = 0.063 determined via 10-fold cross-validation) was applied, and two most discriminative features—Kurtosis and Entropy—were selected for further analysis (Fig 1). The Kurtosis and Entropy values were significantly higher in the PIMA group compared to the non-PIMA group (P < 0.05). In contrast, no statistically significant differences were observed between the two groups in Mean Value, Standard Deviation, Quantile 10, Quantile 25, Quantile 50, Quantile 75, Quantile 90, Skewness, Energy, Variance, Surface Area, Flatness, Elongation, Minor Axis, Major Axis, or High Gray Level Emphasis (P > 0.05, Table 2). Fig 1 illustrates representative radiomic features in a typical case of pulmonary invasive mucinous adenocarcinoma. The chest CT image shows a large patchy area of increased attenuation in the right lower lobe with blurred margins. Localized small cystic areas of low attenuation are visible, while no significant calcification or cavitation is observed.

**Table 2:** CT image omics parameters of the two groups($\overset{―}{x}$±s).

Parameter	PIMA(n = 150)	non-PIMA(n = 150)	*t*	*P*
Mean Value	21.26 ± 2.42	20.58 ± 3.85	1.831	0.068
std.Deviation	3.89 ± 1.02	3.75 ± 0.97	1.218	0.224
Quantile 10	32.25 ± 10.32	30.75 ± 9.42	1.315	0.190
Quantile 25	47.58 ± 8.74	49.51 ± 10.39	1.743	0.080
Quantile 50	110.35 ± 21.38	113.45 ± 18.74	1.335	0.183
Quantile 75	162.25 ± 24.32	160.57 ± 19.31	0.663	0.508
Quantile 90	195.62 ± 13.45	193.47 ± 15.87	1.266	0.207
Skewness	0.042 ± 0.015	0.039 ± 0.024	1.298	0.195
Kurtosis	3.20 ± 0.57	3.05 ± 0.42	2.595	0.010
Entropy	9.18 ± 2.42	6.45 ± 1.89	10.889	<0.001
Energy	0.005 ± 0.003	0.005 ± 0.002	0.000	1.000
Variavce	435.68 ± 98.51	415.67 ± 102.35	1.725	0.086
Surface Area	1321.32 ± 452.39	1385.68 ± 356.78	1.368	0.172
Flatness	0.72 ± 0.15	0.75 ± 0.26	1.224	0.222
Elongation	0.85 ± 0.21	0.89 ± 0.29	1.368	0.172
Minor Axis	11.52 ± 2.69	11.78 ± 2.97	0.795	0.427
Major Axis	16.51 ± 3.22	15.98 ± 2.99	1.477	0.141
High Gray Level Emphasis	365.87 ± 42.68	366.71 ± 46.84	0.162	0.871

### 3.2. Plasma mRNA levels of FoxM1 and Sox9 in the two groups

**Fig 2:**
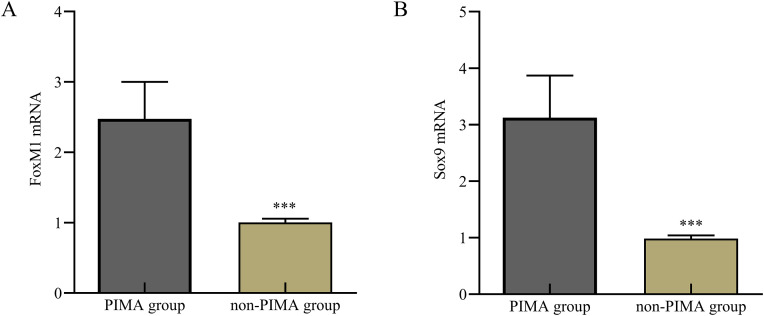
Comparison of plasma FoxM1 and Sox9 mRNA levels between the two group. A: FoxM1 mRNA; B: Sox9 mRNA. Compared with PIMA group, P < 0.001.

The plasma levels of FoxM1 in the PIMA group and the non-PIMA group were 2.48 ± 0.52 and 1.00 ± 0.05, respectively, while the mRNA levels of Sox9 were 3.12 ± 0.74 and 0.99 ± 0.06, respectively. The plasma mRNA levels of FoxM1 and Sox9 in the PIMA group were significantly higher than those in the non-PIMA group (P < 0.05) (Fig 2).

### 3.3. Comparison of CT radiomic parameters and plasma mRNA levels of FoxM1 and Sox9 among patients with different pathological features in the PIMA group

No significant differences were found in kurtosis, entropy, and plasma mRNA levels of FoxM1 and Sox9 when comparing patients with different ages, genders, smoking histories, and lymph node metastasis status (P > 0.05). However, significant differences were observed in kurtosis, entropy, and plasma mRNA levels of FoxM1 and Sox9 among patients with different degrees of differentiation and TNM stages (P < 0.05) (Table 3).

**Table 3:** Comparison of qualitative and quantitative parameters of 3D CT in patients with different prognosis ($\overset{―}{x}$±s).

Pathological feature	n	Kurtosis	Entropy	FoxM1 mRNA	Sox9 mRNA
Age(y)					
<60	62	3.15 ± 0.98	9.21 ± 2.54	2.39 ± 0.75	3.15 ± 0.99
≥60	88	3.24 ± 0.92	9.16 ± 2.35	2.54 ± 0.82	3.10 ± 0.90
*t*		0.574	0.124	1.142	0.321
*P*		0.567	0.901	0.255	0.748
Sex					
male	85	3.18 ± 0.95	9.11 ± 2.51	2.52 ± 0.74	3.16 ± 0.85
female	65	3.23 ± 0.79	9.13 ± 2.43	2.43 ± 0.65	3.07 ± 0.97
*t*		0.343	0.049	0.778	0.604
*P*		0.732	0.961	0.438	0.547
Smoking(cm)					
yes	87	3.19 ± 0.87	9.20 ± 2.68	2.39 ± 0.68	3.18 ± 0.95
no	63	3.21 ± 0.98	9.15 ± 2.74	2.60 ± 0.80	3.04 ± 0.87
*t*		0.132	0.112	1.733	0.923
*P*		0.895	0.911	0.085	0.358
TNM stage					
Ⅰ ~ Ⅱ	82	2.87 ± 0.79	8.27 ± 2.11	2.10 ± 0.65	2.56 ± 0.67
Ⅲ	68	3.60 ± 1.15	10.28 ± 3.10	2.94 ± 0.89	3.80 ± 1.16
*t*		4.590	4.704	6.669	8.177
*P*		<0.001	<0.001	<0.001	<0.001
Degree of differentiation					
high	39	2.65 ± 0.78	8.32 ± 2.42	1.98 ± 0.62	2.44 ± 0.56
medium	57	3.11 ± 0.68	9.21 ± 2.69	2.35 ± 0.75	2.97 ± 0.95
low	54	3.69 ± 0.98	10.57 ± 3.11	2.98 ± 0.87	3.77 ± 1.12
*F*		18.556	7.784	20.640	24.106
*P*		<0.001	<0.001	<0.001	<0.001
Lymphnode metastasis					
yes	105	3.18 ± 0.85	9.16 ± 2.75	2.45 ± 0.67	3.09 ± 0.98
no	45	3.25 ± 0.98	9.23 ± 2.97	2.55 ± 0.76	3.19 ± 1.02
*t*		0.441	0.139	0.804	0.566
*P*		0.660	0.889	0.423	0.572

### 3.4. Correlation between CT image omics parameters, plasma mRNA levels of FoxM1 and Sox9, and pathological features

**Fig 3:**
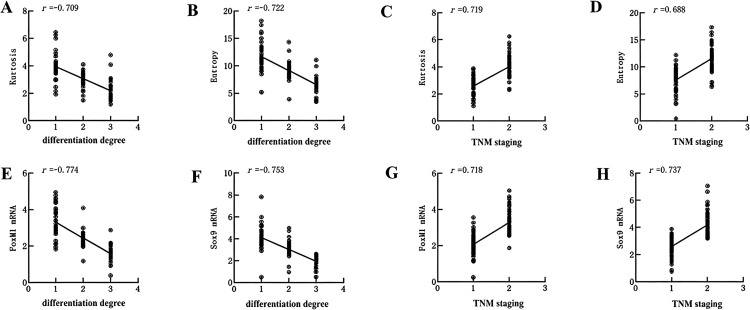
Correlation between Kurtosis, Entropy, plasma FoxM1, Sox9 mRNA levels and TNM staging. A: Kurtosis vs differentiation degree (r = −0.709); B: Entropy vs differentiation degree (r = −0.722); C: Kurtosis vs TNM staging (r = 0.719); D: Entropy vs TNM staging (r = 0.688), **(E)** FoxM1 mRNA vs differentiation degree (r = −0.774); F: Sox9 mRNA vs differentiation degree (r = −0.753); G: FoxM1 mRNA vs TNM staging (r = 0.718), **(H)** Sox9 mRNA vs TNM staging (r = 0.737). TNM staging: Ⅰ ~ Ⅱ = 1, Ⅲ = 2. All correlations are statistically significant (*P* < 0.05).

Spearman correlation analysis showed that kurtosis, entropy, and plasma mRNA levels of FoxM1 and Sox9 were negatively correlated with the degree of differentiation (r = −0.709, −0.722, −0.753, *P* < 0.05), but positively correlated with TNM stage (r = 0.719, 0.688, 0.737, *P* < 0.05) (Fig 3).

### 3.5. The Impact of CT Radiomic Parameters and Plasma FoxM1, Sox9 Levels on PIMA

Using PIMA as the dependent variable (1 = yes, 0 = no), Kurtosis, Entropy, plasma FoxM1 mRNA, and Sox9 mRNA were treated as independent variables (with actual values entered), and logistic regression analysis was performed. The results showed that Kurtosis, Entropy, plasma FoxM1 mRNA, and Sox9 mRNA are all influencing factors of PIMA (*P* < 0.05). See Table 4.

**Table 4:** The Impact of CT Radiomic Parameters and Plasma FoxM1, Sox9 Levels on PIMA.

Variable	*β.*	*SE*	*Wald x* ^*2*^	*RR*	95%*CI*		*P*
					Lower	Upper	
Kurtosis	0.982	0.285	11.881	2.671	1.528	4.669	<0.001
Entropy	0.848	0.215	15.561	2.335	1.532	3.559	<0.001
FoxM1 mRNA	0.352	0.126	7.824	1.423	1.111	1.821	0.005
Sox9 mRNA	1.156	0.198	34.109	3.178	2.156	4.685	<0.001

### 3.6. The value of CT image omics parameters and the plasma mRNA levels of FoxM1 and Sox9 in the differential diagnosis of PIMA

**Fig 4:**
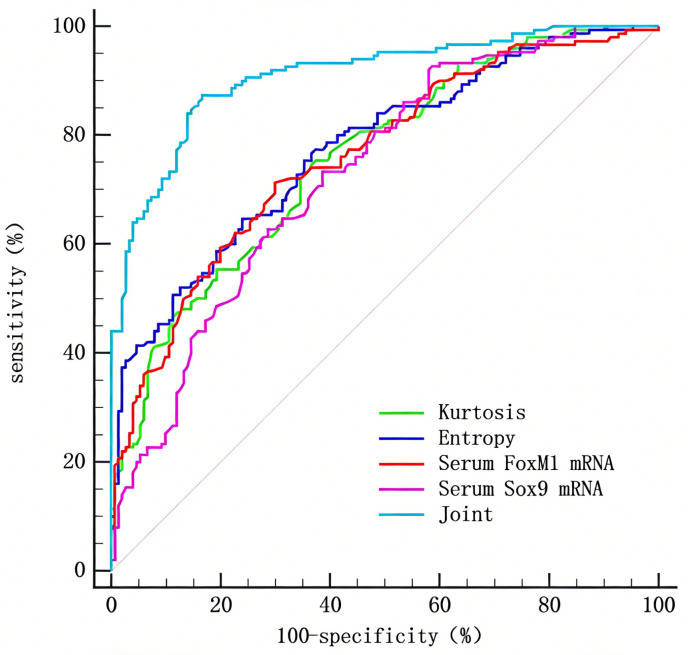
ROC curve of differential diagnosis of PIMA by CT imaging parameters and plasma FoxM1 and Sox9 mRNA levels.

Using the PIMA group dataset as positive control samples and the non-PIMA group dataset as negative samples, variables that showed statistical significance in the univariate analysis (Kurtosis, Entropy, plasma FoxM1 mRNA, and Sox9 mRNA) were included in the multivariate logistic regression analysis. The results demonstrated that all four were independent discriminative diagnostic factors for PIMA (P < 0.05), as shown in Table 4. ROC curves were plotted to evaluate the diagnostic performance of CT radiomic parameters, plasma FoxM1 mRNA, and Sox9 mRNA levels in discriminating PIMA. The results indicated that the combination of Kurtosis, Entropy, plasma FoxM1 mRNA, and Sox9 mRNA levels yielded the largest area under the curve (AUC), which was significantly greater than that of each indicator alone (P < 0.05). See Fig 4 and Table 5. The combined diagnostic model constructed based on multivariate logistic regression analysis showed an AUC of 0.910 (95% CI: 0.872–0.940) for discriminating PIMA. To further evaluate the stability and generalizability of the model, internal validation was performed using the Bootstrap method with 1000 resampling iterations. The calibrated AUC was 0.892, indicating that the model has good predictive performance and clinical applicability.

**Table 5:** Value of CT imaging parameters, plasma FoxM1 and Sox9 mRNA levels in differential diagnosis of PIMA.

Parameter	AUC(*95%CI*)	Cutoff value	Sensitivity(%, *95%CI*)	Specificity(%, *95%CI*)	*P*
Kurtosis	0.758(0.705 ~ 0.805)	>2.77	75.33(67.86 ~ 81.54)	62.67(54.70 ~ 70.00)	<0.001
Entropy	0.775(0.723 ~ 0.821)	>8.40	64.67(56.74 ~ 71.86)	76.00(68.57 ~ 82.13)	<0.001
FoxM1 mRNA	0.762(0.710 ~ 0.809)	>2.11	71.33(63.64 ~ 77.97)	70.00(62.24 ~ 76.76)	<0.001
Sox9 mRNA	0.730(0.676 ~ 0.780)	>2.68	73.33(65.74 ~ 79.76)	61.33(53.35 ~ 68.75)	<0.001
Combination	0.910(0.872 ~ 0.940)		87.33(81.06 ~ 91.74)	83.33(76.55 ~ 88.45)	<0.001

### 3.7. Follow-up Results

**Fig 5:**
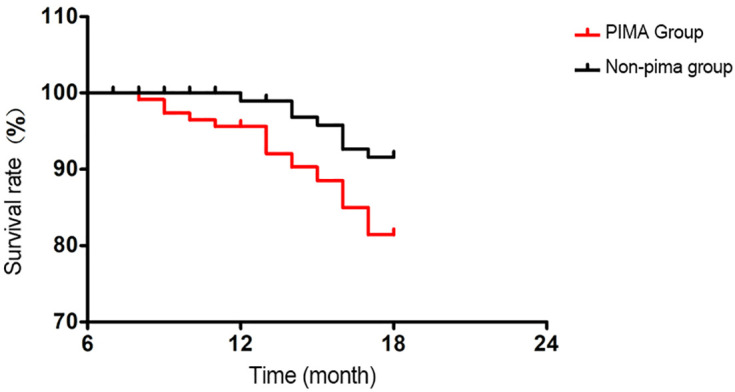
Survival curve.

The median follow-up time was 12 months (range: 6–18 months). The recurrence rate in the PIMA group was 24.67% (37/150), and the metastasis rate was 30.00% (45/150), the mortality rate was 14.67%(22/150). In the non-PIMA group, the recurrence rate was 12.00% (18/150), and the metastasis rate was 16.67% (25/150),the mortality rate was 5.33%(8/150). Statistically significant differences were observed in both recurrence and metastasis rates between the two groups (P < 0.05), see Fig 5.

## 4. Discussion

Previous studies have shown that in clinical practice, PIMA presents different and similar imaging features compared to non-PIMA. Even, the imaging features of PIMA are similar to some CT signs of benign lesions of the lung, which can lead to misdiagnosis or missed diagnosis and delay the treatment opportunity [8]. Yonghui *et al*. [9] reported that isolated PIMA and non-PIMA show differences in CT imaging features, which helped identify clinical PIMA and improve diagnostic accuracy. Based on previous studies, this study introduced imaging omics technology, which is a non-invasive research method. Using a computer, it provides quantitative characteristic parameters from CT images, which have a high predictive value in the identification of benign and malignant lung tumors and can help the diagnosis of lung adenocarcinoma [10]. For example, Chen XM et al. [11] found that CT-omics technology is superior to the lung-RADS model in the identification of benign and malignant pulmonary nodules. Some studies have also found that CT image omics features have a certain value in the differential diagnosis of PIMA and pulmonary lymphoma [12]. This study found that kurtosis and entropy values in the PIMA group were higher than those in the non-PIMA group, and these values were strongly correlated with the degree of differentiation and TNM staging of patients with PIMA. These findings are similar to those reported in previous studies [13–14], indicating that CT radiomic parameters are associated with the malignant progression of lung adenocarcinoma. Kurtosis and entropy are two profoundly important texture parameters. The former primarily describes the distribution pattern of data and is used to measure the peakedness of the distribution; a higher kurtosis value indicates a sharper distribution and is associated with a higher degree of malignancy. The latter is employed to quantify the uncertainty of information, reflecting the randomness and complexity of image pixels. A higher entropy value suggests that the image contains more information and exhibits a more heterogeneous pixel distribution [15].

FoxM1 is a member of the forkhead transcription factor family. It is located in the 12p13-3c region and is composed of 10 exons. Its expression is upregulated in mammalian tissues with high proliferation activity [16]. Previous studies have shown that increased expression of FoxM1 in solid malignant tumors is associated with various biological processes, such as tumor cell proliferation, invasion, migration, and apoptosis [17]. Chunxia *et al.* [18] showed that FoxM1 is upregulated in the plasma and tissues of patients with lung adenocarcinoma, and is associated with the degree of tumor differentiation and lymph node metastasis. Therefore, it is expected to become a diagnostic marker for lung adenocarcinoma. In this study, we found that the plasma mRNA level of FoxM1 in the PIMA group was higher than that in the non-PIMA group. Consistent with the aforementioned study [18], these findings indicate that elevated FoxM1 is associated with the malignancy of lung adenocarcinoma, with higher FoxM1 levels correlating with greater malignant potential. Further analysis revealed that in the PIMA group, FoxM1 mRNA expression was negatively correlated with the degree of differentiation and positively correlated with TNM stage, further supporting the above conclusion. Basic research demonstrated via microarray analysis that FoxM1 is highly expressed in lung adenocarcinoma tissues. Knockdown of FoxM1 inhibited the migration, invasion, and epithelial-mesenchymal transition of lung adenocarcinoma cells by downregulating the expression of metastasis-related proteins. In vivo xenograft experiments showed that knocking down FoxM1 suppressed tumor growth and metastasis in mice [19]. The study by Li PP et al. [20] indicated that FoxM1 serves as an indicator of poor prognosis in lung adenocarcinoma patients, and its knockdown inhibited the migration and proliferation of lung adenocarcinoma cells.

Sox9 is a member of the Sox family, mainly located in the sex-determining region of sex chromosomes, the SRY region. It possesses highly conserved evolutionary characteristics and is involved in physiological functions, such as sex determination, cell proliferation and differentiation, apoptosis, and cartilage differentiation [21]. Recent studies have shown that Sox9 is upregulated in malignant tumors, such as lung cancer, osteosarcoma, and prostate cancer. It is closely associated with pathological features, such as the degree of tumor differentiation and lymph node metastasis, providing a basis for the diagnosis and treatment of malignant tumors [22]. This study found that the plasma mRNA levels of Sox9 in the PIMA group were higher than those in the non-PIMA group, and it had a certain association with the degree of differentiation and TNM staging among patients with PIMA. This finding suggests that Sox9 overexpression is associated with the malignant progression of lung adenocarcinoma. Zhong H et al. [23] reported that Sox9 expression was upregulated in KRAS-driven gene-induced lung adenocarcinoma cells. Sox9 silencing inhibited the infiltration of dendritic cells into lung adenocarcinoma, thereby suppressing the infiltration and function of natural killer cells and exerting antitumor effects. Studies have also found that Sox9 expression is upregulated in lung adenocarcinoma tissues and cells, and Sox9 overexpression can promote the invasion and migration of lung adenocarcinoma cells, while Sox9 knockout inhibited the invasion and migration of lung adenocarcinoma cells [24]. Chengwei et al. [25] reported that downregulation of Sox9 inhibited the proliferation of lung adenocarcinoma cells and induced apoptosis, indicating an important biological role of Sox9 in tumor progression. The combination of Kurtosis, Entropy, plasma FoxM1, and Sox9 mRNA levels demonstrated the highest diagnostic efficacy, with an AUC of 0.910, which was significantly greater than that of any individual indicator alone. These findings suggest that CT radiomic parameters combined with plasma FoxM1 and Sox9 may serve as a novel panel for the diagnosis of PIMA, providing a reference for non-invasive and rapid diagnostic strategies.

Further follow-up of patients in this study revealed that the recurrence and metastasis rates in the PIMA group were significantly higher than those in the non-PIMA group, suggesting that PIMA is associated with greater invasiveness and poorer prognosis. The combination of abnormal expression of radiomic parameters and plasma biomarkers may not only aid in the differential diagnosis of PIMA but also provide important references for prognostic evaluation. Future studies should expand the sample size and extend the follow-up period to further validate the value of these indicators in predicting PIMA recurrence and metastasis.

This study employed a rigorous radiomic feature selection process. Initially, intraclass correlation coefficient (ICC) analysis was used to ensure the reproducibility of feature extraction. Subsequently, Least Absolute Shrinkage and Selection Operator (LASSO) regression—a method suitable for high-dimensional feature selection—was applied to screen the initially extracted 18 features. Ultimately, two most representative radiomic features, Kurtosis and Entropy, were selected, effectively avoiding overfitting and enhancing model reliability. Based on this, a multivariate logistic regression diagnostic model was constructed by incorporating plasma FoxM1 mRNA and Sox9 mRNA levels. This model demonstrated excellent diagnostic performance in the development set (AUC = 0.910). After internal validation using the Bootstrap method, the calibrated AUC remained at 0.892, indicating good stability and generalizability of the model, thereby providing a more reliable tool for the non-invasive diagnosis of PIMA.

The results of this study showed that the radiomic parameters Entropy and Kurtosis were significantly higher in the PIMA group than in the non-PIMA group and were closely associated with poorer differentiation and more advanced TNM staging. An increase in Entropy reflects greater irregularity and randomness in the grayscale distribution of the image, which at the biological level may indirectly indicate high intratumoral heterogeneity—manifested as significant variations in cell morphology, tissue architecture, necrotic/cystic areas, and distribution of mucinous components. PIMA is characterized by the production of large amounts of mucus by tumor cells. These mucinous areas appear as hypodense regions on CT images and are interwoven with solid tumor components and blood vessels, forming a complex and heterogeneous texture that leads to elevated Entropy. Meanwhile, Kurtosis describes the deviation of the grayscale distribution from a normal distribution, reflecting the “peakedness” or “flatness” of the distribution. The increased Kurtosis in PIMA suggests the presence of more extreme grayscale values (very high or very low), likely corresponding to densely packed solid components (high density) and extensive mucinous pools or cystic areas (very low density). This polarized histological composition results in a “heavy-tailed” grayscale distribution, thereby contributing to higher Kurtosis. These findings align with the clinical recognition of PIMA as a more aggressive subtype of lung adenocarcinoma.

This study also found that high expression levels of FoxM1 and Sox9 mRNA were significantly correlated with imaging heterogeneity in PIMA. FoxM1 promotes cell proliferation and invasion, potentially leading to disorganized tumor architecture and complex textural patterns on imaging. Sox9, as a downstream effector of the KRAS pathway, mediates immune evasion and may influence mucinous stroma formation and tumor growth patterns, collectively shaping the characteristic imaging phenotype. Compared with previous radiomic studies such as those in AJR 2023, Heliyon 2024, and BMC Cancer 2023, which were based solely on morphological features, this study innovatively integrates plasma molecular biomarkers, providing a molecular functional interpretation of the imaging phenotype. This approach represents a transition from “imaging features” to “biological mechanisms,” enhancing the interpretability and clinical relevance of the model.

Patients with pulmonary invasive mucinous adenocarcinoma (PIMA) exhibit poorer survival outcomes compared to those with invasive non-mucinous adenocarcinoma (non-PIMA), characterized by significantly higher rates of short-term recurrence and metastasis. These findings align with the higher malignant potential and more aggressive biological behavior of PIMA. Previous work from this study demonstrated that the PIMA group exhibited higher radiological heterogeneity (Entropy) and elevated expression levels of oncogenes (FoxM1 and Sox9). These characteristics collectively contribute to an increased risk of disease progression. Although the follow-up period in this study remains relatively short, the results clearly untangle the adverse prognostic trend associated with PIMA, underscoring the importance of recognizing PIMA as a distinct clinicopathological entity that warrants aggressive treatment and close monitoring.

In conclusion, CT-based radiomic parameters and plasma FoxM1 and Sox9 mRNA levels in PIMA patients are associated with the degree of differentiation and TNM stage. Kurtosis, Entropy, and plasma FoxM1 and Sox9 mRNA levels can be used for the differential diagnosis of PIMA, and their combined use demonstrates high diagnostic value. Radiomics technology enables high-throughput feature extraction and data analysis from medical images, providing detailed information about organs and tissues. When combined with plasma biomarkers, it improves diagnostic accuracy and offers a non-invasive diagnostic tool for PIMA, greatly expanding the application of non-invasive diagnostics in the practice of precision medicine.

This study has several limitations. First, this was a single-center retrospective study without external validation, which may limit the generalizability of the proposed model. Future multicenter prospective studies are warranted to further validate these findings. Second, the tumor size criterion (>3 cm) was applied only to the PIMA group, which may have introduced selection bias between groups. This factor might partially influence radiomic feature differences. Additionally, the median follow-up period of 12 months may be insufficient to draw definitive conclusions regarding the long-term prognosis and recurrence of PIMA. Longer follow-up periods are needed to assess the clinical outcomes more comprehensively.

## Supporting information

S1 File Raw data. (XLSX)
